# The association between blood glucose levels and matrix-metalloproteinase-9 in early severe sepsis and septic shock

**DOI:** 10.1186/s12950-016-0122-7

**Published:** 2016-04-22

**Authors:** Gul R. Sachwani, Anja K. Jaehne, Namita Jayaprakash, Mark Kuzich, Violet Onkoba, Dione Blyden, Emanuel P. Rivers

**Affiliations:** Henry Ford Hospital, 2799 W Grand Blvd, Detroit, MI 48202 USA

**Keywords:** Matrix-Metalloproteinase-9, MMP-9, Septic shock, Sepsis, Cytokines, IL-8, ICAM-1, Glucose

## Abstract

**Background:**

Hyperglycemia is a frequent and important metabolic derangement that accompanies severe sepsis and septic shock. Matrix-Metalloproteinase 9 (MMP-9) has been shown to be elevated in acute stress hyperglycemia, chronic hyperglycemia, and in patient with sepsis. The objective of this study was to examine the clinical and pathogenic link between MMP-9 and blood glucose (BG) levels in patients with early severe sepsis and septic shock.

**Methods:**

We prospectively examined 230 patients with severe sepsis and septic shock immediately upon hospital presentation and before any treatment including insulin administration. Clinical and laboratory data were obtained along with blood samples for the purpose of this study. Univariate tests for mean and median distribution using Spearman correlation and analysis of variance (ANOVA) were performed. A *p* value ≤ 0.05 was considered statistically significant.

**Results:**

Patients were grouped based on their presenting BG level (mg/dL): BG <80 (*n* = 32), 80–120 (*n* = 53), 121–150 (*n* = 38), 151–200 (*n* = 23), and > 201 (*n* = 84). Rising MMP-9 levels were significantly associated with rising BG levels (*p* = 0.043). A corresponding increase in the prevalence of diabetes for each glucose grouping from 6.3 to 54.1 % (*p* = 0.0001) was also found. As MMP-9 levels increased a significantly (*p* < 0.001) decreases in IL-8 (pg/mL) and ICAM-1 (ng/mL) were noted.

**Conclusion:**

This is the first study in humans demonstrating a significant and early association between MMP-9 and BG levels in in patients with severe sepsis and septic shock. Neutrophil affecting biomarkers such as IL-8 and ICAM-1 are noted to decrease as MMP-9 levels increase. Clinical risk stratification using MMP-9 levels could potentially help determine which patients would benefit from intensive versus conventional insulin therapy. In addition, antagonizing the up-regulation of MMP-9 could serve as a potential treatment option in severe sepsis or septic shock patients.

## Background

Hyperglycemia is a frequent and important metabolic derangement that can occur in severe sepsis or septic shock. The up-regulation of several hormones (glucagon, growth hormone, catecholamines, and glucocorticoids), inflammatory mediators (interleukin (IL)-1, IL-6, Intercelluar Adhesion Molecule (ICAM-1), and tumor necrosis factor (TNF)-alpha) accompanies the development of hyperglycemia [1, 2]. Elevated blood glucose (BG) has been associated with an increase in oxidative stress, as reflected by increase in oxygen free radical (O_2_^.^) generation [3]. In addition to oxidative stress, there is also inflammatory stress, because O_2_^.^ activates a number of redox-sensitive pro-inflammatory transcription factors, such as nuclear factor (NF)-κB, activator protein–1 (AP-1), early growth response–1 (EGR-1), and hypoxia-inducible factor–α [3, 4].

Insulin administration is not only important in modulating glycemic control, but it also aids in antagonizing the pro-inflammatory effects of elevated blood glucose levels in critically illness. Insulin has been shown to suppress 3 important inflammatory mediators: ICAM–1, monocyte chemoattractant protein–1 expression, and NF-κB binding in human aortic endothelial cells in vitro [3, 5, 6].

Several studies attempting to elucidate the molecular mechanisms of hyperglycemia and sepsis have revealed elevated levels of Matrix-Metalloproteinase-9 (MMP-9) [7–12]. MMP-9 is a pro-inflammatory biomarker belonging to a family of zinc-containing endoproteinases that are implicated in chronic cell remodeling, migration, adhesion, and apoptosis. In humans, MMPs consist of a family of approximately 26 members including the collagenases (MMP1, 8, 13), stromelysins (MMP3, 10), matrilysins (MMP7, 26), and gelatinases (MMP2, 9) [13, 14]. In the setting of sepsis there is a sequestration of polymorphonuclear neutrophils (PMNs) at the site of infection which is modulated through the processing of various cytokines and chemokines, in particular IL-8 and ICAM-1. MMP-9 is released by activated PMNs at the site of infection resulting in tissue injury, and further perpetuation of the vicious cycle of inflammation and tissue destruction [15].

Despite the abundance of literature in animal and human models describing the association of elevated MMP-9 levels with two distinct entities such as hyperglycemia and sepsis, the link between BG and MMP-9 levels in humans with severe sepsis and septic shock has not been described. The aim of our study was to examine the clinical and pathogenic link between MMP-9 and BG levels in patients with early severe sepsis and septic shock.

## Methods

### Patients

After obtaining approval by the Henry Ford Health System Institutional Review Board (IRB) we conducted a prospective observational study in a tertiary care, university affiliated hospital. Patients included in the study presented to the emergency department (ED) from 1997 to 2000 and were diagnosed with severe sepsis or septic shock. Blood samples (serum and plasma) were collected, spun, and stored at −80 °C upon presentation prior to the administration of any therapy including insulin. Patients’ baseline characteristics, including basic demographics, comorbidities, presenting clinical data, and Acute Physiology and Chronic Health Evaluation II (APACHE II) admission scores were recorded. Patients were grouped by glucose levels based on recommendations in the literature for glycemic control in critical illness during the study period.

### MMP-9, IL-8, & ICAM-1 immunoassays

Biomarker assays were independently performed by Biosite, Inc. (Alere), San Diego, Calif. Assays were performed using immunometric (sandwich) assays performed with NeutrAvidin-coated 384-well block microtiter plates (Pierce Biotechnology, Inc.; Rockford, Ill) and a Genesis RSP 200/8Workstation (Tecan US, Inc., Durham, NC). Each sample was tested in duplicate. Before performing any assays, biotinylated primary antibody was diluted in an assay buffer containing 10 mmol/L tris(hydroxymethyl)amino-methane HCl (pH 8.0), 150 mmol/L sodium chloride, 1 mmol/L magnesium chloride, 0.1 mmol/L zinc chloride, and 10 mL/L polyvinyl alcohol (9Y10 kDa). The concentration of biotinylated antibody was predetermined by titration. The primary antibody (10 2 L per well) was added to the plates and incubated. After washing, 10 g/L bovine serum albumin and 1 g/L sodium azide were added to the plate wells that were then incubated at room temperature. Next, the plates were washed three times with borate-buffered saline containing 0.02 % polyoxyethylene (20) sorbitan monolaurate (BBS-Tween).

Ten-microliter aliquots of each sample were added to each plate well, and the plates were incubated. Following this incubation, the plates were washed three times and alkaline phosphatase-conjugated antibody (10 2 L per well) was added to each plate well and further incubated. The concentration of the alkaline phosphatase-conjugated antibody was predetermined to ensure a linear profile in the dynamic range of interest. After additional incubation, the plates were washed nine times with BBS-Tween. AttoPhos substrate (S1011; Promega Corporation, Madison, Wis), a fluorescence-enhancing substrate previously diluted in AttoPhos buffer (S1021; Promega), was then added to aid in the measurement of the activity of antibody-conjugated alkaline phosphatase bound in each well. The plates were then scanned in a fluorometer (Tecan Spectrafluor; Tecan US, Inc.) using an excitation wavelength of 430 nm and an emission wavelength of 570 nm. Each well as scanned six times at 114-second intervals, and the rate of fluorescence generation was calculated.

### Statistical methods

Descriptive statistics were used to analyze comorbidities, baseline clinical data, organ dysfunction scores, and mortality. All continuous variables are expressed as mean ± standard deviation (STDE). We also performed a univariate test for means and median distribution using Spearman correlation and analysis of variance (ANOVA). A *p* value < 0.05 was considered statistically significant. IBM SPSS v19.0 for Windows was used for all statistical computations.

## Results

### Patients

A total of 230 patients with severe sepsis and septic shock presenting to the ED were included in the study. Patients were grouped based on presenting BG levels as follows: <80 mg/dL: *n* = 32; 80–120 mg/dL: *n* = 53; 121–150 mg/dL: *n* = 38; 151–200 mg/dL: *n* = 23; >201 mg/dL: *n* = 84 (Table. 1). The age of the overall study population was 66 ± 17.2 years and the gender distribution was 53:47 (Male: Female). The mean APACHE II score of all patients was 22 ± 7. The baseline demographics for each BG group are shown in Table 1. The age, race, and gender distributions were similar among the BG groupings. The rates of pre-existing conditions such as hypertension, chronic obstructive pulmonary disease (COPD), and congestive heart failure (CHF) were similar among the BG groups. However, there was an increase in the prevalence of diabetes mellitus (DM) as BG levels increased (*p* = 0.0001). The prevalence of DM was 6.3 % (*n* = 2) in the low BG group (<80 mg/dL) and increased to 54.1 % (*n* = 46) in the high BG group (>201 mg/dL). Although not statistically significant, patients in the low BG group (<80 mg/dL) and 80–120 mg/dL group had higher rates of pre-existing liver failure (31.3 % and 33 % respectively) when compared to other BG groups. In addition, patients in the low BG group were noted to have a higher incidence of pre-existing renal failure (18.8 %). A significant difference in APACHE II score on ED admission was observed between patients in the various BG groupings (*p* = 0.019). APACHE II scores were highest in the <80 mg/dL BG group (23.8 ± 6.9) and in the 151–200 mg/dL group (24 ± 7.2).

**Table 1:** Patient basic demographics and comorbidities per Glucose Grouping

*Variable*	All patients	<80 mg/dL	80–120 mg/dL	121–150 mg/dL	151–200 mg/dL	>201 mg/dL	*P* value
n	230	32	53	38	23	84	
Age (years ± SD)	66 ± 17	63 ± 17	59 ± 25	70 ± 15	70 ± 13	66 ± 16	0.08
Gender (M:F) %	53:47	44:56	68:32	50:50	56:44	49:51	0.18
Race, *n* (%)							
AA	201(87)	31(97)	47(89)	32(84)	20(87)	71(85)	0.56
Caucasian	26(11)	1(3)	6(11)	5(13)	3(13)	11(13)	
Other	3(1)			1(3)		2(2)	
Pre-existing conditions, *n* (%)							
Hypertension	153(67)	22(69)	29(54)	25(64)	14(61)	63(74)	0.2
COPD	34(15)	5(16)	6(11)	10(26)	3(13)	10(12)	0.3
CHF	82(36)	12(37)	22(41)	12(31)	10(43)	26(31)	0.6
Diabetes Mellitus	57(25)	2(6)	3(6)	2(5)	4(17)	46(54)	0.0001
Liver failure	63(27)	10(31)	18(33)	8(20)	5(22)	22(26)	0.6
Renal failure	33(14)	6(19)	6(11)	7(18)	3(13)	11(13)	0.8
APACHE II, mean ± SD	22 ± 7	24 ± 7	19 ± 6	22 ± 7	24 ± 7	21 ± 7	0.019

*M* Male, *F*, Female, *AA* African American, *COPD* Chronic obstructive pulmonary disease, *CHF* Congestive heart failure

### BG levels and basic clinical data upon ED presentation

Table 2 summarizes baseline vital signs and routine laboratory findings. The mean heart rate (HR), systolic blood pressure (SBP), and respiratory rate (RR) were similar among the groups. Patients with <80 mg/dL had lower body temperatures (34.8 °C ± 3.4 °C; *p* = 0.017) and higher central venous pressures (CVP; 8.95 mmHg  ± 9.5 mmHg; *p* < 0.0001) when compared to the other BG groupings. The mean glucose levels (mg/dL) for each BG group were as follows: <80: 46 ± 25; 80–120: 100 ± 12; 121–150: 133 ± 8; 151–200: 173 ± 14; and >201: 543 ± 428. ANOVA revealed a statistically significant difference in the mean BG values (*P* < 0.0001). Lactate levels were noted to be highest in the <80 mg/dL (10.1 mg/dL ± 5.1 mg/dL). An increase in lactate levels were noted in the subsequent BG groupings (range 5.5 mg/dL ± 4.7 mg/dL to 7.3 mg/dL ± 4.2 mg/dL; *p* < 0.002). Consistent with the presence of infection, the >201 mg/dL BG group had the highest WBC count (16 mm^3^ ± 8.8 mm^3^), culture positivity (80 %) and bacteremia rates (42.4 %).

**Table 2:** Patient clinical and routine laboratory findings upon presentation

*Variable (mean ± SD)*	<80 mg/dL	80–120 mg/dL	121–150 mg/dL	151–200 mg/dL	>201 mg/dL	*P* value
Temperature, °C	34.8 ± 3.4	36.5 ± 2.9	35.8 ± 2.5	36.3 ± 3.1	36.8 ± 2.5	0.017
HR, beats/min	106 ± 30	120 ± 26	115 ± 33	117 ± 34	120 ± 29	0.22
SBP, mmHg	106 ± 37	107 ± 36	96 ± 27	97 ± 33	113 ± 35	0.079
CVP, mmHg	8.95 ± 9.5	9.3 ± 8.5	4.3 ± 8.9	2.8 ± 6.2	1.96 ± 6.2	<0.0001
Glucose, mg/dL	46 ± 25	100 ± 12	133 ± 8	173 ± 14	543 ± 428	<0.0001
Lactate, mg/dL	10.1 ± 5	5.5 ± 4.7	5.9 ± 3.5	7.1 ± 5.1	7.3 ± 4.2	0.002
WBC, mm^3^	12 ± 10	11 ± 8	15 ± 10	15 ± 8	16 ± 9	0.008
Anion Gap, mM/L	24 ± 8	19 ± 9	20 ± 5	20 ± 6	23 ± 8	0.003
ScvO_2, %_	61 ± 24	49 ± 18	49 ± 15	48 ± 10	55 ± 10	0.2
BUN, mg/dL	44 ± 26	41 ± 36	50 ± 29	53 ± 29	46 ± 33	0.53
Creatinine, mg/dL	3 ± 2	2 ± 2	3 ± 2	3 ± 2	2 ± 2	0.22
Positive Blood Culture, *n* (%)	22(69)	42(78)	30(77)	16(70)	68(80)	0.7
Bacteremia, n (%)	13(41)	15(28)	14(36)	8(35)	36(43)	0.5

*°C* Degrees Celsius, *HR* Heart Rate, *SBP* Systolic Blood Pressure, *CVP* Central Venous Pressure, *WBC* White Blood Cell, *ScvO*_*2*_ Central Venous Oxygen Saturation, *BUN* Blood Urea Nitrogen

### Association between BG, MMP-9, IL-8, and ICAM-1

**Fig. 1:**
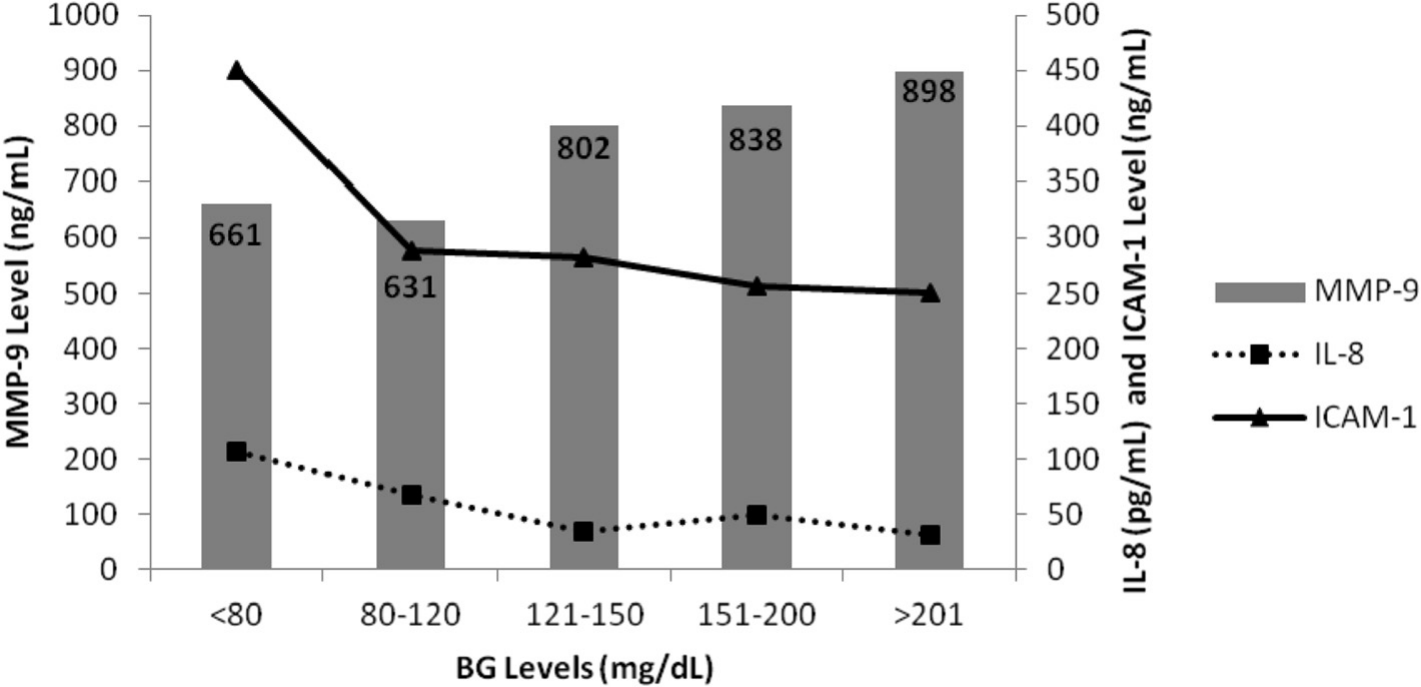
Association of increasing MMP-9 (grey bars) levels with increasing blood glucose (BG) levels. Inverse proportional association of IL-8 (┈■┈) and ICAM-1 (**—**▲**—**) with MMP-9 levels

Figure 1 demonstrates the relationship of BG with MMP-9, IL-8, and ICAM-1 levels. Rising MMP-9 levels were significantly associated with rising BG levels (*p* = 0.043). For the various BG (mg/dL) groupings MMP-9 levels (ng/mL) were as follows: <80: 661, 80–120: 631, 121–150: 802, 151–200: 838, and >201: 898. As MMP-9 levels increased with rising BG levels a statistically significant decrease in IL-8 and ICAM-1 levels was noted (*P* < 0.0001). For each BG (mg/dL) group the IL-8 (pg/mL) levels were as follows: : <80: 107, 80–120: 67, 121–150: 34, 151–200: 49, and >201: 31. Similarly, a significant inverse relationship was observed for ICAM-1 levels (ng/mL) for each BG group: <80: 450, 80–120: 289, 121–150: 282, 151–200: 256, and >201: 250.

## Discussion

To our knowledge this is the first study to establish a significant association between BG levels and MMP-9 levels in patients with severe sepsis or septic shock. In addition, we also demonstrate that as BG increased an inverse relationship between MMP-9 and IL-8 levels, and MMP-9 and ICAM-1 levels was observed.

Currently, it is well-known that in severe sepsis and septic shock patients, hyperglycemia develops due to a combination of several factors: 1) Insulin clearance is increased leading to a reduction in insulin-mediated glucose uptake; 2) Stress induced elevation in plasma levels of counter-regulatory hormones, such as catecholamines, glucagon, cortisol, and growth hormone. These hormones lead to hyperglycemia due to muscle glycolysis and lipolysis, and subsequent gluconeogenesis and glycolysis in the liver; 3) Hyperlactatemia due to glycolysis in muscle caused by the counterregulatory hormones and cytokines, sometimes referred to as the “lactate shuttle” 4) Insulin resistance which could be due to a defective GLUT4 transporter and to the deleterious effects of pro-inflammatory cytokines such as IL-1, IL-6, and TNF-α [16–18].

It has been demonstrated via various significant in vivo and in vitro experimental studies that in the setting of acute or chronic hyperglycemia (diabetes mellitus), glucose can induce the increase of pro-inflammatory transcription factors such as NF-kB, AP-1, and EGR-1. The MMP-9 promoter region has been described to contain responsive elements to the aforementioned transcription factors and thus up-regulating MMP-9 transcription and activity [5, 10, 19, 20]. Our results may suggest a possible up-regulation of MMP-9 in the setting of both acute and chronic hyperglycemia. The majority of patients with BG ranging from 121 mg/dL to 200 mg/dL had increased MMP-9 levels most likely secondary to an acute increase in their BG levels in the setting of critical illness. This is inferred from the study data as the prevalence of DM in patients with a BG ranging from 121 mg/dL to 200 mg/dL was only 5 % to 17 %. Consistent with the literature, the highest MMP-9 level (898 ng/mL) was noted in patients in the high BG (>201 mg/dL) group. This group had the highest mean BG level (543 mg/dL ± 428 mg/dL) upon presentation to the ED and it also had the highest prevalence of DM (56 %).

High MMP-9 levels have been identified in patients in severe sepsis or septic shock. In addition to the up-regulation of MMP-9 gene transcription secondary to pro-inflammatory factors, MMP-9 levels are increased in the setting of sepsis due to the release of MMP-9 by activated PMNs at the site of infection causing tissue injury and further perpetuating the vicious cycle of inflammation and tissue destruction [15, 21]. The significant rise of MMP-9 across the BG groupings in this study cannot solely be attributed to acute (stress) or chronic hyperglycemia. In our study patients with low BG (<80 mg/dL) or normoglycemia (80–120 mg/dL) still had elevated MMP-9 levels (661 ng/mL and 631 ng/mL respectively) leading us to the conclusion that the MMP-9 elevation in these two groups is probably due to the increase in neutrophil activation rather than stress response or chronic hyperglycemia [22].

The rise in MMP-9 levels in patients with severe sepsis or septic shock has been described to occur very early in the disease process. In an experimental study involving human subjects Albert et al., demonstrated that MMP-9 levels peaked 2 h after the administration of bacterial lipopolysaccharide (LPS) [23]. Nakamura et al., provided the first report of elevated plasma MMP-9 levels in 20 patients with septic shock. They were able to demonstrate that plasma MMP-9 concentrations were significantly higher in the non-surviving patients with septic shock than in the surviving patients with septic shock [24]. Since then, various studies have corroborated the increase of MMP-9 levels in patients with severe sepsis or septic shock and their poor outcomes [11, 12, 25–27]. In one of our previous study in which we described the natural history of circulatory biomarker activity in the most proximal phases of severe sepsis and septic shock we also noted that MMP-9 levels peaked early (6 h) after presentation [28].

The rise of MMP-9 in septic patients can be due to: 1) genetic up-regulation and 2) release by activated neutrophils. Together with MMP-9 levels we measured the levels of two biomarkers (IL-8 and ICAM-1) intimately involved with neutrophil function. IL-8, a member of the CXC chemokine family has been reported to be elevated and play a role in the pathophysiology of sepsis because of its ability to attract, activate and degranulate neutrophils [29, 30]. In addition, the expression of ICAM-1 (a cell surface glycoprotein) is increased on the cell surface of neutrophils allowing it to attach and migrate through the endothelium during sepsis [31, 32]. Our results demonstrate the existence of an inverse relationship between MMP-9 and IL-8 as well as MMP-9 and ICAM-1 as BG levels increased. A possibility behind the inverse relationship noted between MMP-9 and IL-8 is that MMP-9 is known to cleave a specific site of IL-8 molecular structure rendering a variant IL-8 molecule which is 10 to 30 fold more potent in neutrophil activation [33, 34]. We theorize that our assay provided us with the measurement of the native IL-8 molecule and not the variant IL-8 form which we conceptually believe to be elevated. The aforementioned observation describes the relationship between IL-8 and MMP-9 from a proteonomic and metabolomic point of view. However, the possibilities of genomic links between IL-8 and MMP-9 needs to be further investigated. To explain the relationship between ICAM-1 and MMP-9 is more challenging as there is a scarcity of studies describing this relationship. In the field of oncology MMP-9 has been shown to proteolytically cleave the extracellular domain of ICAM-1 leading to its release from the cell surface [35]. Thus, it would lead us to believe that ICAM-1 levels should in fact rise as MMP-9 levels increase. Our observation contrast this finding. A possible explanation to the rise of MMP-9 and decrease of ICAM-1 levels may be due to the direct effect of insulin on such biomarkers. Aljada et al., demonstrated that insulin inhibits the expression of ICAM-1 in human aortic endothelial cells mediated by an increase in nitric oxide (NO) release and nitric oxide synthase (NOS) expression [36]. On the other hand, Fischoeder et al., described that insulin was a potent inducer of MMP-9 activity in primary human monocytes [37].

The early pathogenic link between elevated MMP-9 levels and elevated BG levels make it plausible to consider that early inhibition or removal of this biomarker may have therapeutic potential. Antibiotics, such as the tetracyclines inhibit MMPs, not only by chelating the zinc and calcium ions but also by affecting the induction of the MMP genes. The therapeutic administration of tetracyclines over prolonged periods of time may cause undesirable side effects, such as gastrointestinal disturbances and emergence of antibiotic resistance. A recent pilot study reported a low side effect profile of doxycycline (tetracycline) based on sub-antimicrobial concentrations dosing (100 mg intravenous (IV) daily, followed by 50 mg IV once daily for 2 days). However, their results showed no effect on MMP-9 levels [38]. The chemically modified tetracyclines (CMT), which lack antibacterial activity but retain the associated anti-inflammatory properties of tetracyclines are the preferred MMP inhibitor because they offer several advantages over conventional tetracyclines. The CMT have been proposed to induce no gastrointestinal side effects or toxicities, attain higher concentrations in plasma, and cross the blood–brain barrier and blood-retina barrier [39]. In an animal model for sepsis, Maitra et al., demonstrated that MMP-9 levels together with glutamic oxaloacetic acid transaminase (GOT), glutamic pyruvic transaminase (GPT), and nitric oxide (NO) were decreased when septic rats were pre-treated with CMT 24 h and 1 h prior to cecal ligation and puncture (CLP) [40]. They also reported that the 24 h mortality rates for CLP rats were much higher in the untreated group (30 %) when compared to the CMT pretreatment group in which no deaths were reported. In this case, the prophylactic administration of CMT in the setting of sepsis has shown to effectively decrease MMP-9 levels and improve survival. However, the more clinical relevant question is to determine the appropriate treatment window in which CMTs can be administered in order to decrease MMP-9 levels, improve survival, and decrease the sequela of sepsis (i.e. ARDS; liver failure). Halter et al., performed an animal study to test the hypothesis of a treatment window during which CMT could be administered in the setting of sepsis. Even though they did not specifically measure MMP-9 levels, they discovered that CMT given at 6 and 12 h after CLP improved survival and lung injury rates when compared to the group that received CMT at 24 h. Therefore, Halter’s group concluded that CMT can be administered as late as 12 h after septic insult [41].

Another alternative that has shown to decrease circulating levels of MMP-9 and improve survival in the setting of sepsis is the use of hemoperfusion using polymixin B immobilized on fibers (PMX-F). Nakamura et al., demonstrated that hemoadsorption therapy using PMX-F columns decreased the level of circulating MMP-9 and improved the survival rate of the patients with septic shock. They hypothesized that hemoperfusion therapy with PMX-F attenuated the increase in plasma MMP-9 concentrations in patients with septic shock by reducing plasma endotoxin [24].

Much controversy has been generated in regards to glycemic control since the landmark article of van den Berghe et al., which demonstrated decreased mortality in critically ill patients treated with intensive insulin therapy [42]. Since then, several RCTs and meta-analyses have emerged revealing no mortality benefits in critically ill patients initiated on an intensive insulin therapy [43–48]. In fact, the NICE-SUGAR trial demonstrated an increase in mortality in patients undergoing intensive glucose control (BG level target ≤ 110 mg/dL) when compared to the group treated with a BG target of 180 mg/dL [49]. In order to achieve adequate BG control in septic patients a different approach may need to be undertaken. This study shows a close association between elevated pro-inflammatory cytokines and the development of hyperglycemia in severe sepsis and septic shock patients. Therefore, adjusting treatment based on the level of pro-inflammatory cytokines may be an option for adequate glucose control [50, 51]. Nakamura et al., demonstrated a significant positive correlation between a pro-inflammatory cytokine (IL-6) and BG level in septic patients. Their results showed that the rate of successful glucose control decreased with an increase in the blood IL-6 level and that in the failed glucose control group the insulin dose per 100 kcal energy intake was higher [51]. Keeping in mind the concept of hypercytokinemia and hyperglycemia in septic patients together with the results reported by Fischoeder et al., in which insulin was found to be a potent inducer of MMP-9 activity in primary human monocytes [37], we hypothesize that MMP-9 levels may serve as a marker to help stratify septic patients to an intensive versus conventional insulin therapy group. We believe that patients with elevated MMP-9 and BG levels may be stratified to a conventional insulin therapy group (BG target of 180 mg/dL) and insulin should be judiciously used to avoid further release of MMP-9 as this will subsequently increase the rate of extracellular matrix (ECM) remodeling and its sequelae.

Notable strengths of this study include the large number of patients recruited and the ability to measure MMP-9 levels very early in the disease process. The MMP-9 levels were measured upon presentation to our ED before any treatment (i.e. antibiotics, intravenous fluids, or insulin) was initiated. Together with the conventional therapeutic strategies used for septic patients, antagonizing the up-regulation of MMP-9 could serve as a potential treatment option. In order to establish the use of MMP-9 as a possible biomarker for glucose control and also as a therapeutic target in septic patients further prospective trials are required. In addition, more studies are also required to explain the inverse relationship noted between IL-8 and ICAM-1 with MMP-9.

## Conclusion

This prospective observational study in humans demonstrates a significant and early association between MMP-9 and BG levels in patients with severe sepsis and septic shock. Neutrophil affecting biomarkers such as IL-8 and ICAM-1 are noted to decrease as MMP-9 levels increase. Risk stratification using MMP-9 levels could potentially help determine which patients would benefit from intensive versus conventional insulin therapy. In addition, antagonizing the up-regulation of MMP-9 could serve as a potential treatment option in severe sepsis or septic shock patients.
